# Anxiety, depression, stress, worry about COVID-19 and fear of loneliness during COVID-19 lockdown in Peru: A network analysis approach

**DOI:** 10.3389/fpubh.2022.946697

**Published:** 2022-09-09

**Authors:** José Ventura-León, Renato López-Jurado, Emilia Porturas, Irina León-Mostacero, Sherily Edith Canchanya-Balbin

**Affiliations:** ^1^Facultad de Ciencias de la Salud, Universidad Privada del Norte (UPN), Lima, Peru; ^2^Organización MEPPCi, Pontificia Universidad Católica del Perú (PUCP), Lima, Peru; ^3^Organización MEPPCi, Universidad Nacional Mayor de San Marcos (UNMSM), Lima, Peru

**Keywords:** clinical disorders, worry about COVID-19, fear of loneliness, COVID-19 lockdown, network analysis

## Abstract

This study aims to examine the relationships between symptoms of anxiety, depression, stress, worry about COVID-19 and fear of loneliness during COVID-19 lockdown in Peru using network analysis. There were 854 participants aged 18 to 50 years (Mean = 36.54; *SD* = 9.23); 634 females (74.20%) and 220 males (25.80%), who completed the Generalized Anxiety Disorder Scale (GAD-7), Patient Health Questionnaire (PHQ-9), Perceived Stress Scale (PSS-10), Preoccupation with COVID-19 Contagion (PRE-COVID-19), Brief Scale of Fear of Loneliness (BSFL). A partial unregularized network was estimated through the *ggmModSelect* function. Expected influence (EI) and bridging EI values were calculated to identify central symptoms and bridging symptoms respectively. The results reveal those two symptoms of depression—stress and anxiety—were the most central symptoms in the network. Depressive symptoms are at the same time the most comorbid and it is shown that there are no differences in the network when compared between those who left home and those who did not leave home during lockdown. Depressive symptoms are concluded to be central and bridging in the network and interconnected with some symptoms of stress and anxiety. These findings may be important to understand the experience of COVID-19 lockdown in Peru.

## Introduction

The COVID-19 pandemic led to the implementation of extraordinary health measures, example of which was mandatory social isolation or lockdown. This event consisted of prolonged periods of time without leaving the home and it has been shown to have repercussions on mental health. Specifically, people with no history of clinical disorders presented symptoms of distress and those with a pre-existing diagnosis showed a worsening of their symptomatology (1). In this regard, Rossi et al. (2) in a large-scale study (*N* = 18,147) showed that at the beginning of the pandemic there were severe symptoms of depression (20.8%), anxiety (7.3%) and perceived stress (22.9%). In fact, depressive (27.6 %) and anxious (32.6 %) symptoms were higher than in previous years where they barely reached 4% (3, 4). A similar situation occurred during the Severe Acute Respiratory Syndrome (SARS) epidemic when lockdown produced changes in people's anxiety and stress, increasing depression and suicide rates (5). The impact was such that 1 year after the epidemic people still showed symptoms of distress and stress (6).

Stress is defined as an individual's actual or anticipated experience of an adverse, unpredictable and uncontrollable situation (7). In this sense, the cognitive appraisal theory indicates that stress is an individual's evaluative response to a situation greater than the personal resources (8). According to its persistence over time, it can be categorized as acute if the stressor stimulus is transitory, and chronic if the difficulties perceived by the individual are maintained for a prolonged period of time (9). Stress can appear in different ways and have adverse consequences on health. Thus, during COVID-19 lockdown, there is evidence that perceived stress was directly related to insomnia, loneliness and anxiety (10–12).

Anxiety is conceived as the set of physical and psychological manifestations, which can be diffuse, prolonged or intense, that are attributed to a stimulus of real or imaginary danger (13). It is strongly related to fear since they are both experienced in a dangerous situation (14). At low levels, it can be considered as an adaptive response to potential threats (15), and at high levels, it de-structures the individual's personality (15, 16). Thus, anxiety symptoms during lockdown showed a strong relationship with other clinical conditions (12) and its presence in the pandemic gave rise to a new construct such as Worry about COVID-19 contagion.

Worry about COVID-19 contagion can be understood as thoughts about the probability of becoming infected by coronavirus, which in turn affect mood and the ability to perform activities and cause a problem for the individual (17). Studies associated with infectious diseases show that the assessment of the risk of becoming infected is greater in early periods of the pandemic (18). Some studies have indicated that people more worried about the pandemic presented a greater probability of adopting preventive behaviors (19). Furthermore, they experienced a deterioration in their mental health and life satisfaction and an increase in generalized anxiety, stress (20) and depression (21–23).

Depression is a mood disorder that consists of a set of physical and mental symptoms, such as sleep difficulties, reduced appetite, low mood, reduced interest in pleasurable activities, among others (24). It is one of the considerable factors in understanding the risk of suicide, which has increased in recent years (25, 26). Likewise, during health emergency, these situations have also had relevance (5). These data manifest a possible increase in depression figures during the COVID-19 pandemic. They are estimated to reach a prevalence of 27% in the general population (11) and its implications are greater when associated with feelings of loneliness (27).

Loneliness, from a cognitive perspective, is understood as a negative feeling that is the result of unmet needs for bonding and social activities (28). It is usually experienced as a feeling of not belonging and misunderstanding or lack of companionship (29, 30). This causes psychological ailments, expressed in boredom or emotional emptiness (31), and generates fear of finding oneself alone. Fear of loneliness can be defined as worry responses and avoidance behavior to the experience of being alone (32). During lockdown, people who were isolated showed a significant increase in loneliness (27); specifically, young people and adults (33, 34). In these populations, loneliness impacted interpersonal relationships, acquisition of addictive behaviors, depressive symptoms, and decreased subjective wellbeing (27, 28, 35).

The above proves that anxiety, depression, stress, worry about COVID-19 contagion and fear of loneliness may be relevant variables to be examined during COVID-19 lockdown in Peru. In fact, specialized literature states that the onset of a depressive episode is usually preceded by stressful events that alter the individual's wellbeing (36, 37) and provoke anxiety responses (38). A tentative explanation is that stress comprises physiological and behavioral responses that lead the individual to the evaluation of real or perceived threats (39). At this point, stress proves its relationship with anxiety by sharing cognitive or evaluative factors of a dangerous event. These responses increased during the pandemic; especially, in people who worried about their health (40).

Under this idea, Endler (41) states that there is an interconnection between anxiety and stress by personal variables (vulnerability, heredity, cognitive style, trait anxiety) and interaction with stressful situations (discomfort, pain, trauma) or biological ones indicating that these clinical disorders share neural circuits (39). In this sense, during lockdown, stress and depression were symptoms that presented greater connection and strength of relationship (42–44). Likewise, both symptoms were associated with loneliness during confinement (27); especially in people who were alone, between 18 to 30 years old and with low family income (33). At the same time, anxiety proves to be a clinically relevant disorder during lockdown, but unstable to resample procedures in network analysis (12). Perhaps, this is because individuals present a specific anxiety trait such as worry about being infected with COVID-19 that is associated with stress, anxiety (20) and depression (21–23).

The study has strong implications for public health because the strategies of total confinement implemented worldwide occurred in countries where health systems had serious structural problems and may have collapsed (45). This is the case of Peru, where confinement measures were strict. At the Latin American level, confinement revealed the lack of confidence of governments to contain the virus (46). For instance, in countries where focused confinement was implemented, such as Chile, or only social distancing measures were established, such as Uruguay, there was a better localization of the virus, more trust in citizens and better response of the health system to a health emergency (47). Other variables that influenced the implementation of confinement were governments led by extremist political parties and the absence of scientific criteria in those countries (48). At this point, it was not considered that confinement could cause changes in the behavior of the individual. For example, recent research conducted during confinement is showing that the perception of social isolation is a risk factor for the individual's emotional alteration (49) that may predispose them to depression and feelings of loneliness (50, 51). The latter, especially, has a greater impact on stress-related cognitions and behaviors during pandemic (50).

In this regard, this study aims to: (a) identify symptoms of anxiety, depression, stress, worry about COVID-19 Contagion and fear of loneliness in a sample of people during the time of lockdown decreed by the Peruvian government; (b) identify bridging nodes; and (c) compare symptoms according to staying or leaving home during lockdown.

## Methods

### Participants

There were 854 participants aged 18–50 years (Mean = 36.54; *SD* = 9.23); 634 females (74.20%) and 220 males (25.80%). The sample size was estimated *a priori* considering the formula *P*(*P*-1)/2, where *P* is the number of parameters or edges (52) resulting in a minimum of 528 observations. In such sense, the recommended minimum was exceeded. The selected method of sampling was non-probabilistic of a type purposive or judgement sampling, as a group of participants was deliberately selected (53). This was because other methods of sampling were too complex as a result of restrictions imposed by the Peruvian government due to the COVID-19 pandemic. Within other characteristics, 538 (63%) reported leaving their home one or more times a day during lockdown and 316 (37%) did not leave their home (see Table 1).

**Table 1:** Sociodemographic variables.

**Variables**	***f***	**%**
Sex		
Female	634	74.20
Male	220	25.80
Street		
No	316	37.00
Yes	538	63.00
City		
Lima	673	78.80
Province	181	21.20
Annoyed by isolation		
No	762	89.20
Yes	92	10.80
The lockdown has affected their family's finances		
No	202	23.70
Yes	652	76.30
Do you live with an older adult?		
No	459	53.70
Yes	395	46.30
Have you had flu symptoms?		
No	567	66.40
Yes	287	33.60

Street: Did you go out during the lockdown period?

### Instruments

*Generalized Anxiety Disorder Scale* [GAD-7, (54)] composed of seven items with response alternatives from 0 to 3 (0 = No day, 1 = Several days, 2 = More than half of the days, 3 = Almost every day). It is a unidimensional measure that assesses generalized anxiety through worry symptoms. High scores indicate greater anxiety. It has excellent internal consistency (Cronbach's α = 0.92).

*Patient Health Questionnaire* [PHQ-9; (55)] composed of nine items with response alternatives from 0 to 3 (0 = No day, 1 = Several days, 2 = More than half of the days, 3 = Almost every day). It is a unidimensional measure of depressive symptoms, with higher scores indicating greater severity of depression. It has good validity and reliability properties (Cronbach's α = 0.89).

*Perceived Stress Scale* [PSS-10; (56)] in the Spanish version of (57), composed of ten items, with Likert-type response alternatives ranging from 0 to 4 (0 = Never, 1 = Almost never, 2 = Occasionally, 3 = Often, 4 = Very often). It is a unidimensional scale that measures the individual's perception of everyday situations. It has inverse items (4, 5, 7 and 8). High scores indicate a higher degree of perceived stress. It has good validity properties and acceptable reliability (ω = 0.68).

*Worry about COVID-19 Contagion* [WCOVID-19; (17)] composed of six items with response alternatives from 1 to 4 (1 = never or rarely to 4 = almost all the time). It is a unidimensional scale that assesses worry about COVID-19 contagion. High scores indicate a more frequent worry about contagion. It has good validity and reliability properties (ω = 0.90).

*Brief Scale of Fear of Loneliness* [BSFL; (32)] composed of five items with response alternatives from 0 to 4 (0 = Never, 1 = Rarely, 2 = Sometimes, 3 = Almost always, 4 = Always). It is a unidimensional scale that assesses worry, feelings of abandonment and avoidance about being alone. High scores indicate greater fear of loneliness. It has good validity and reliability properties (ω = 0.91).

### Procedures

The instruments were applied collectively using a *Google form* link, which was shared through some social networks (e.g., Facebook and WhatsApp). In this regard, a methodology based on data collection by internet [Internet Mediated Research, IMR; (58)] was taken into consideration. The work described was carried out in accordance with the Code of Ethics of the World Medical Association (Declaration of Helsinki). Therefore, prior to the instruments, the participants responded to an informed consent form explaining the objective of this study, the confidentiality of their information and data processing. Following the acceptance to the research, the participants answered a sociodemographic form to provide information about their age and sex. The collection was carried out during the lockdown decreed by the Peruvian government between 03-31-2020 and 08-06-2020, which was severe during March, but became less severe by the middle of the year. It is worth noting that there was no time limit to respond to the online form; however, on average people responded in 15 min.

### Data analysis

Statistical analyses were performed using the R programming language in its RStudio environment (59). Data analysis was followed using psychological network techniques (52). Specifically, we followed the recommendations by Fried et al. (60) for network analysis: (a) network estimation; (b) network stability review; (c) comparison of two networks by subgroups. The data are available through a link in the Availability of data and material section.

### Previous steps

Previously, an examination of redundant nodes was carried out since it has been highlighted as an important issue in psychological networks (61). It has been noted that they can distort network measures and centrality indices such as expected influence (62).

### Network estimation

The estimation was performed with a non-regularized partial network through the *ggmModSelect* function since the objective was to examine central symptoms and connection bridges. For such purposes, this estimator is optimal (63) because it adds and subtracts edges until minimizing the Bayesian information criterion [BIC; (64)]. Due to the ordinal nature of the variables, partial Spearman correlations which establish the correlation between two symptoms were used after controlling for the other variables in the network (52).

For the interpretation of the network, it should be considered that each node (symptom) is connected to other nodes by edges (lines). The thickness of the edges indicates the strength of the correlation, and the green shade refers to positive correlations while the red shade, to negative ones (52). The network figure was implemented considering the Fruchterman-Reingold algorithm through the *spring* command. In this algorithm, the strongest correlations are located in the center and the weakest ones are in the periphery (65).

The first of the two centrality indices that were estimated is the Expected Influence [EI; (66)]. This index is useful when the nodes (symptoms) have the same interpretation—that is, high scores indicate greater severity of the symptom—and can be considered as an index of “importance.” The second centrality index is the Expected Bridge Influence (EBI), which reflects the strength between a node from a community of nodes of a disorder and one of another community of nodes (67). It is worth mentioning that measures of betweenness and closeness were not estimated because they are not recommended for psychological studies (68).

### Network stability

The accuracy of the edge weights was examined by bootstrapping technique with the *bootnet* package, that consists of repeatedly estimating a model with the sampled data and estimating a value for the edges. This allows the calculation of 95% bootstrap confidence intervals (CI), whose amplitude denotes the stability/instability of the edges (69). Additionally, the correlation stability coefficient (CS), which indicates the proportion of cases to be removed, was calculated. Before at least 95% of 1,000 bootstrap correlations of the true and resampled centrality indices are <0.70. The CS should not be below 0.25 and preferably above 0.50 (52).

### Comparison of two networks

Finally, two sub-networks (going out and not going out during lockdown) were compared using the NCT library (70). The NCT uses a two-tailed permutation test in which the difference between the two groups (going out and not going out) is calculated across 100 replicates for each randomly regrouped individual. The null hypothesis assumes that both groups are equal at the 0.05 significance level.

## Results

### Previous assumption

Preliminarily, the assumption of node redundancy was reviewed, and it was found that the items PSS7, BSFL3, GAD6, PHQ3 were reiterative in the network. Therefore, they were removed from the network estimation.

### Estimate and accurance in the network

**Figure 1:**
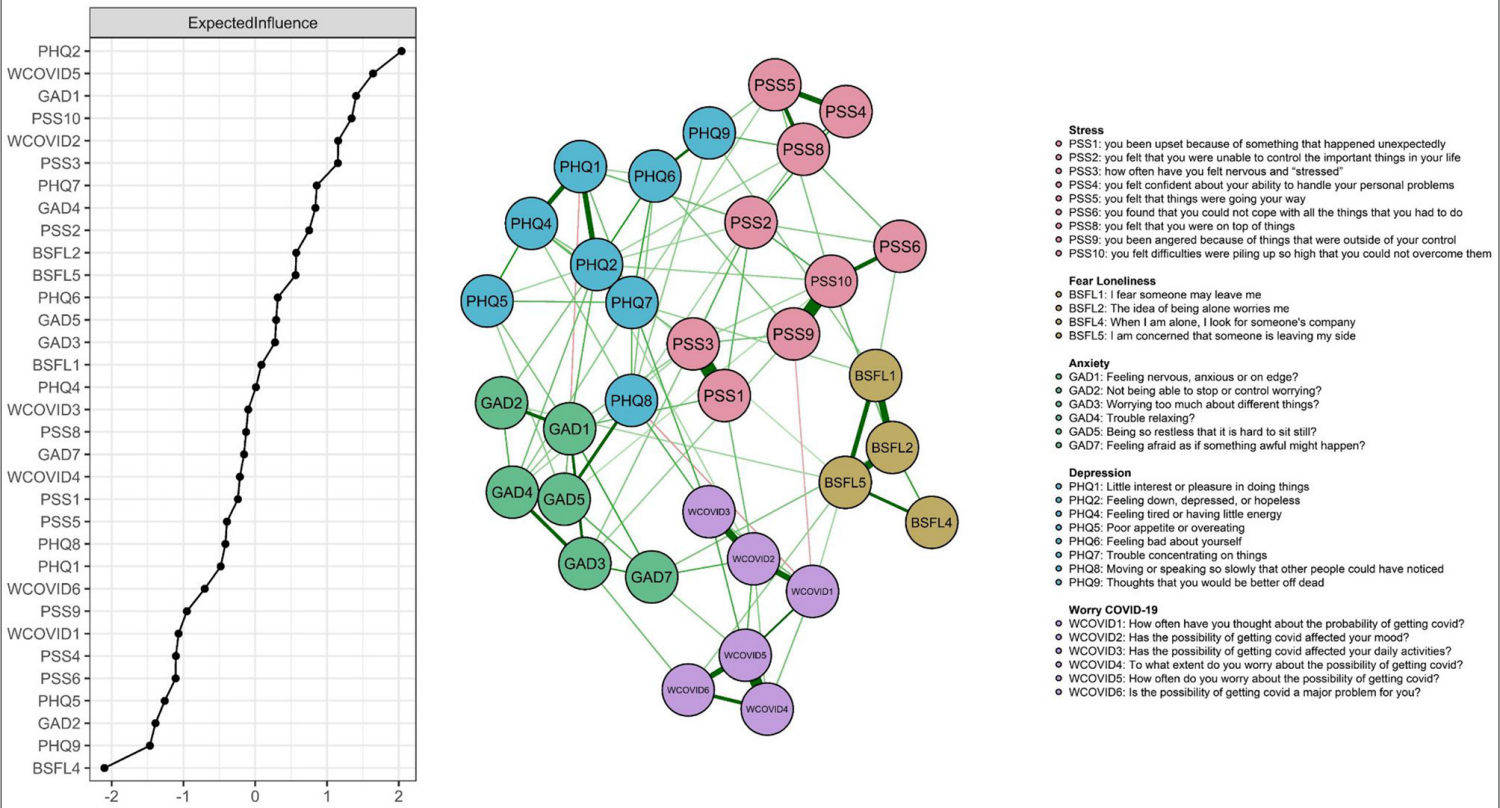
Network model of anxiety, depression, stress, worry about COVID-19 and fear of loneliness during COVID-19 lockdown in Peru. Expected influence statistics are shown in the left panel.

**Figure 2:**
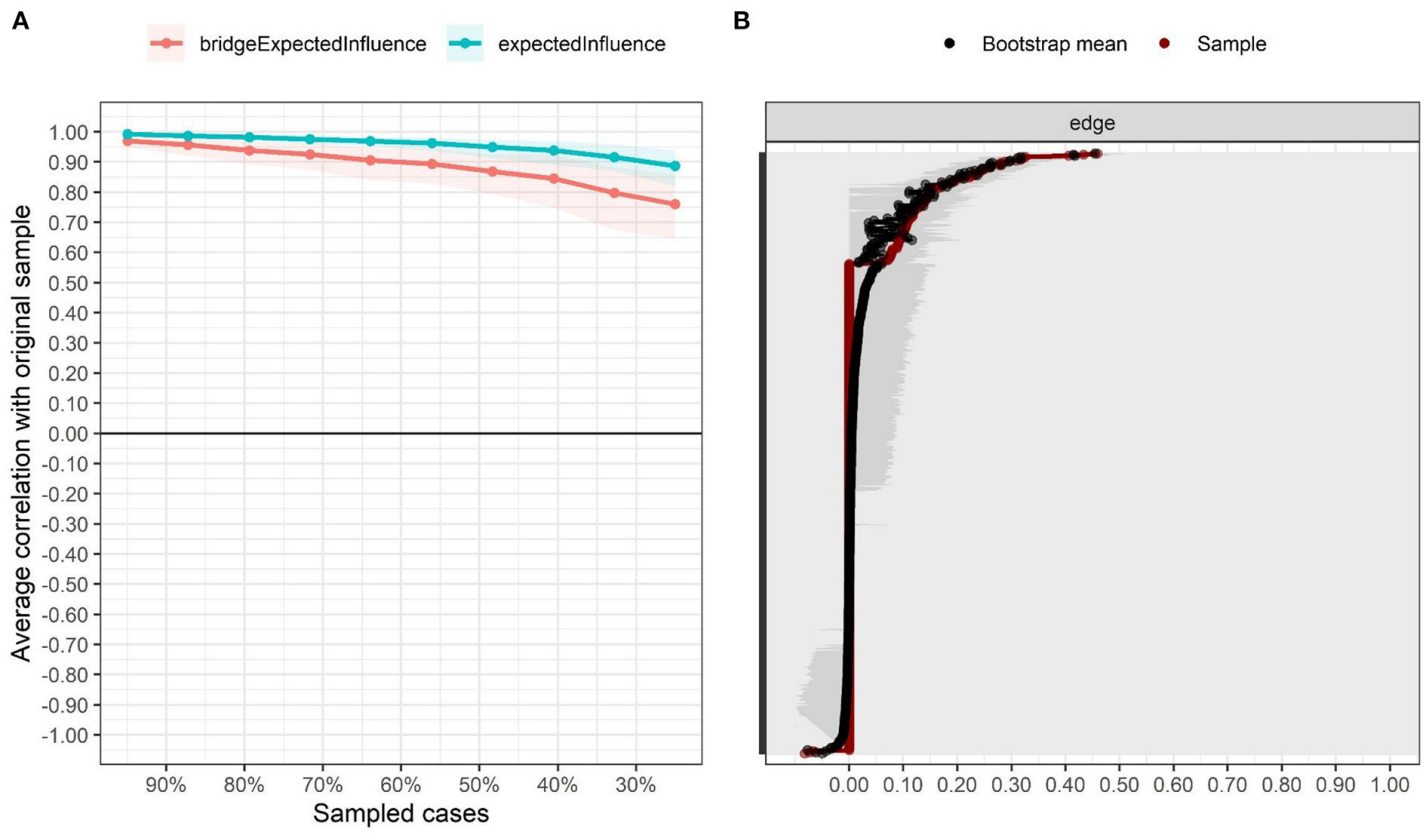
Stability and precision of the estimated network. **(A)** Stability of centrality indices, **(B)** Precision of network edges.

The *ggmModSelect* network for data collected during lockdown are presented in Figure 1. Out of 37 possible edges, only 33 were entered into the final model due to redundancy criteria. The symptoms of each theoretical community (BSFL, GAD, PHQ) were clustered closer to each other. Greater remoteness was found among the stress symptoms. Also, confidence intervals are estimated to examine edge-weight accuracy (see Figure 2).

On the other hand, the EI centrality statistics also shown in Figure 1 demonstrate that symptoms PHQ2, PHQ7, PSS3 and GAD5 present greater interconnectedness in the network, as evidenced by their high EI values. Similarly, the stability statistics confirm their robustness (CS coefficient = 0.75). Thus, robust inferences can be drawn from them (see Figure 2).

**Figure 3:**
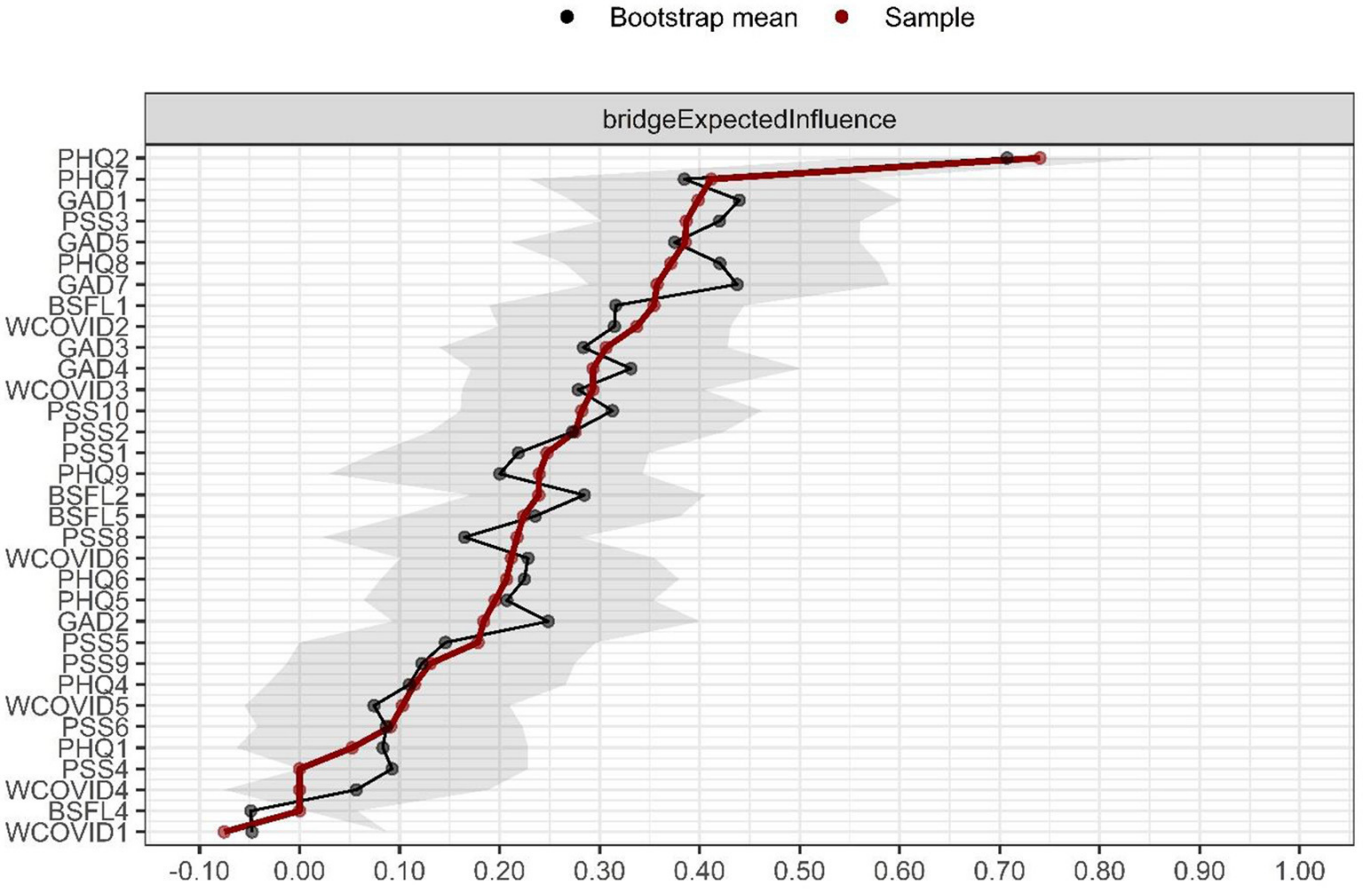
Expected Bridge Influence (EBI). Values are given as Z-scores, with higher values indicating more influential nodes in the network.

In Figure 3, the EBIs are presented. The symptom PHQ2 is observed to be “*you have felt down, depressed, or hopeless”* and PHQ7 “*you have had difficulty concentrating on certain activities, such as reading the newspaper or watching television.”* It indicates that in the PHQ community, these symptoms have stronger connections with the other components of the PSS. Another bridging symptom is GAD1, “*you have felt nervous, anxious, or on edge”* which interconnects with the PSS community. Also, the stability of the bridges was robust (CS coefficient = 0.67).

### Comparison of networks

**Figure 4:**
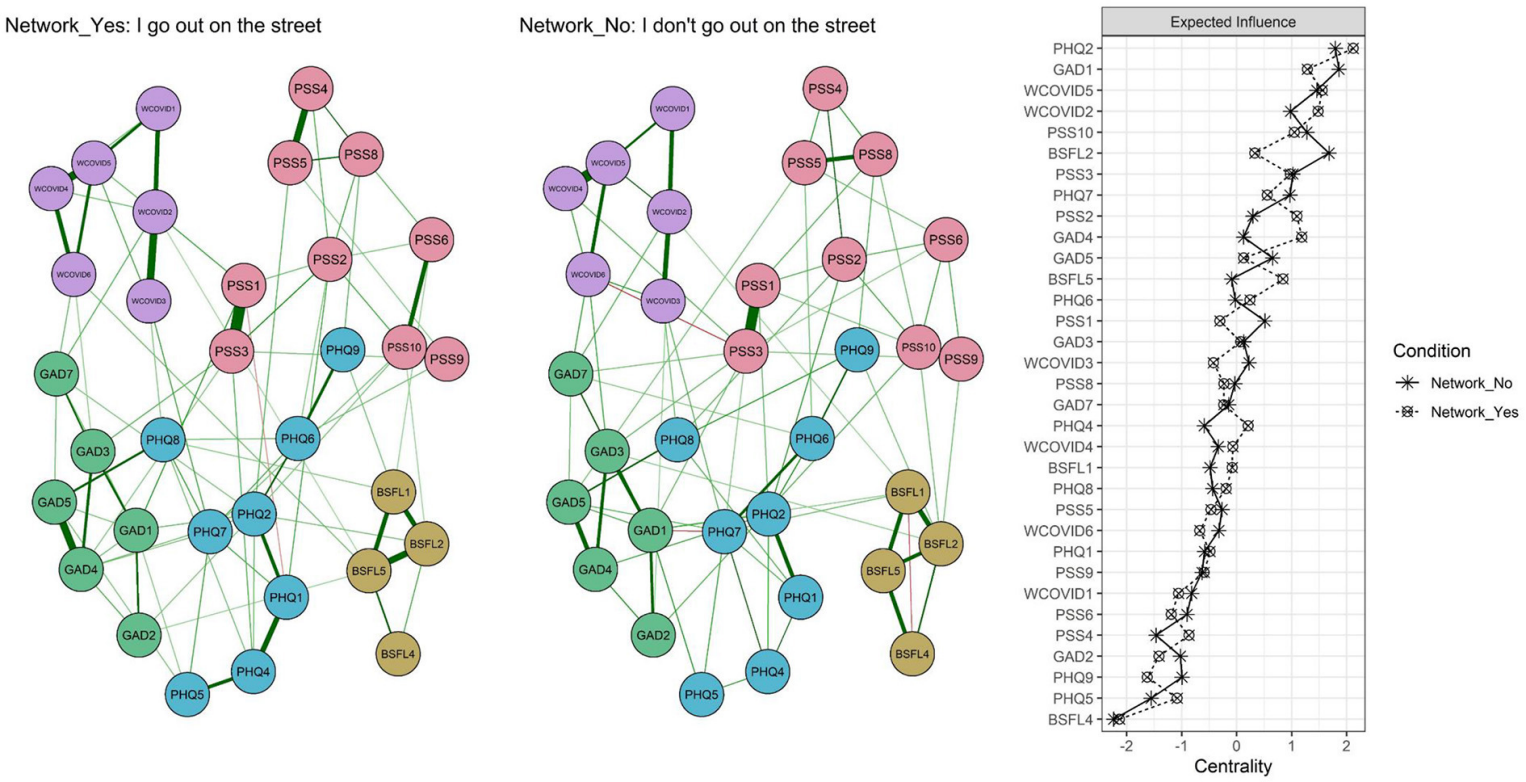
Networks relating to going out or not during lockdown and centrality measure.

Figure 4 shows that at a general level the networks are invariant (M = 0.22; *p* = 0.87) and that the level of connectivity is identical (S = 0.73; *p* = 0.11). Similarly, when the relationship between adjacent matrices is tested with a Pearson correlation calculation, they prove to be similar (*r* = 0.76). However, at a local level, the greatest differences were observed in BSFL2 (“The idea of being alone worries me”), where the EI value was higher in the group that did not go out during lockdown, and in GAD4 (“I have had difficulty to relax”), whose EI was higher in those who did go out.

## Discussion

This study is the first to examine the relationships between anxiety, depression, stress, worry about COVID-19 and fear of loneliness during COVID-19 lockdown in Peru, by a network analysis. The construction of the network of partial correlations allowed examining the connections between symptoms. In this sense, the first objective was to identify the central symptoms in the network. The four nodes with the highest centrality in the network were PHQ2 (“*You have felt down, depressed or hopeless”*), PHQ7 (“*You have had difficulty to concentrate on certain activities, such as reading the newspaper or watching television”*); PSS3 (“*You have felt nervous or stressed”*) and GAD5 (“*You have felt so restless that you have not been able to sit still”*). The first two belong to the depression scale, while the others belong to the stress and anxiety scales, respectively. These results indicate that depressed mood, concentration difficulties, perception of stress and restlessness are central symptoms in the lockdown experience. Symptoms of loneliness fear were not relevant in the network possibly because, during the lockdown, people were with their families and the fear of loneliness was not active (32). A similar situation could have occurred with Worry about COVID-19 Contagion, that by having a greater number of participants in the sample who did not leave home even once, there is a greater willingness in the participants to develop safety behaviors against contagion (20).

The findings demonstrate that there is an interconnection between depressive feelings and stress (PHQ2, PHQ7, PSS3) in Peru during COVID-19 confinement. These results are consistent with previous studies that establish that depression and stress play an important role in the comparison of two temporal networks during COVID-19 (42). In fact, other studies conducted during confinement have pointed out the relationship between social isolation and affective symptoms. It was demonstrated that the level of perceived isolation became more distressing during confinement when there was an absence of physical activity in the individual (49). This may occur when the individual perceives that their personal resources are being overwhelmed by the stressful event (e.g., confinement). As a result, they make a set of unfavorable evaluations (8) that causes a decrease in the individual's well-being and predisposes them toward depressive symptoms (36, 37). On the other hand, detecting a symptom of anxiety (GAD5) is not strange because Zavlis et al. (12) pointed out that, despite the lack of good stability in resampling procedures, anxiety is a central symptom during the COVID-19 pandemic;. In this study, stability of anxiety symptoms was obtained. The explanation for this discrepancy can be found when applying anxiety tests in non-clinical populations. In this scenario, there is a tendency to low scores and anxiety becomes an adaptive factor (15) that does not destructure the personality of the individual (16). It leads, as well, to consider other factors, such as cognitive styles and psychological vulnerability of the individual (41).

A second objective was to identify bridging nodes; those that connect symptom clusters across clinical disorders. The strongest symptoms were PHQ2 (“*You have felt down, depressed or hopeless”*) and PHQ7 (“*You had had difficulty to concentrate on certain activities, such as reading the newspaper or watching television”*), both within the depression scale. These results are related to a previous study that compared depression before and after the peak of the COVID-19 outbreak, where it is highlighted that depressive affect is the central node (51). The results indicate that depressed mood and concentration difficulties are important aspects of depressive disorders. It is evidenced as well that in other health emergencies they have had relevance (5) and their implications are greater when associated with feelings of loneliness. Interestingly, there is a study where concentration problems were observed to emerge as a bridging symptom in seven of thirteen networks while depressive mood emerged in six (71). This suggests that certain symptoms may become transdiagnostic symptoms in clinical disorders (72). Also, it is one of the possible explanations for the increased prevalence of depression during the COVID-19 pandemic (11).

A third objective was to compare symptoms according to staying at home or going out during the lockdown. The results indicate that there are no global differences between the networks and that the adjacent matrices are similar. Despite this, differences are evident in the centrality of the symptom BSFL2 (“*The idea of being alone worries me”*), where the EI value was higher in the group that did not go out during lockdown. These results are consistent with a previous study which showed that loneliness had a greater impact on stress-related cognitions and behaviors during the pandemic (50). These results could be reasonable because lockdown signified a change in human interactions and a need in people for affiliation or belonging (27, 32). Another symptom with strong variance in centrality was GAD4 (“*You have had difficulty to relax”*) in favor of those who left home. A tentative explanation is that a possible COVID-19 infection caused difficulties to relax and a situation of constant tension in those who left home. This is due to the excessive care taken to avoid infection, exemplified by measures such as physical distancing and the use of alcohol gel and masks.

The results have important implications for public health because the implementation of a total confinement measure was subject to the avoidance of the collapse of health systems, the lack of government confidence to contain COVID-19 (45–47), governments with extremist parties in office and the lack of science in some countries (48). Thus, this research provides scientific evidence regarding the psychological effects that confinement had on the Peruvian population. In particular, the study demonstrates that the most central symptoms of the network are related to depression. These findings support models that emphasize the role of depression prevention in the framework of health emergencies, because of its implication on suicide rates (5). Also, its symptoms may be maintained even up to a year after the epidemic event occurred (6). In addition, the study suggests to give relevance to the interaction between depression, stress and anxiety through public policies that encourage the promotion of healthy habits. Physical activity, for instance, became a protective factor during confinement (49). In this way, remote health services (e.g., online or smartphone therapy) should be designed to encourage support networks such as the search for institutions, friends or family members that provide empathy and solidarity to the person. This may be consistent when observing that depression was substantially increased by the COVID-19 pandemic (11) and that it may be higher if the individual has feelings of loneliness (27). Secondly, it is worth noting that the role of perceived stress during lockdown has been shown to be directly related to loneliness, anxiety and insomnia (10–12). Also, the experience of being isolated affected the individual's mental health as demonstrated in a study at the beginning of the pandemic where depression, anxiety and perceived stress are relevant (2). In fact, these clinical disorders proved a worsening of people with a preexisting clinical diagnosis or the presence of distress in people without history (1).

Despite the interesting findings, the study has some limitations. Even though the sample size was sufficient under the formula *P*(*P*-1)/2, other estimation methods based on simulation (52) might suggest a larger number of participants. It should be noted, though, that collection during the months of the study was quite restricted to non-governmental institutions. In addition, the selection of participants was purposive, which reduces the ability to generalize. However, it was the only way to collect data as the collection was conducted during the lockdown and random sampling was complex. Another limitation is related to the difficulty of being able to remotely follow up with respondents or identify them; aspects that are typical of online data collection (58).

## Conclusion

In conclusion, the results indicate that some symptoms of depression, anxiety and stress demonstrate greater interconnectedness in the network. This suggests to focus on depressive symptoms to improve the mental health of people who went through COVID-19 lockdown. The strongest bridging nodes in the network were also identified to be those related to depression In addition, there are no substantial differences between those who went out and did not go out during lockdown, except for node BSFL2 (“The idea of being alone worries me.”). These symptoms may be important to understand how the experience of COVID-19 confinement affected the mental health of Peruvians. According to previous studies, this effect is known to be maintained even up to a year after the health emergency (6). These aspects will be important during the coming years to understand the psychological sequelae of confinement and the COVID-19 pandemic, as they try to forewarn of the responses that should be taken by public health entities in the face of an upcoming pandemic.

## Data availability statement

The original contributions presented in the study are included in the article/supplementary materials, further inquiries can be directed to the corresponding author.

## Ethics statement

The studies involving human participants were reviewed and approved by Universidad Privada del Norte Ethics Committee. The patients/participants provided their written informed consent to participate in this study.

## Author contributions

JV-L, RL-J, EP, IL-M, and SC-B contributed to conception and design of the study. JV-L organized the database, performed the statistical analysis, and critical review of the latest version of the manuscript prior to submission. RL-J, EP, IL-M, and SC-B wrote the first draft of the manuscript. All authors contributed to manuscript revision, read, and approved the submitted version.

## Conflict of interest

The authors declare that the research was conducted in the absence of any commercial or financial relationships that could be construed as a potential conflict of interest.

## Publisher's note

All claims expressed in this article are solely those of the authors and do not necessarily represent those of their affiliated organizations, or those of the publisher, the editors and the reviewers. Any product that may be evaluated in this article, or claim that may be made by its manufacturer, is not guaranteed or endorsed by the publisher.
